# Connecting firm's web scraped textual content to body of science: Utilizing microsoft academic graph hierarchical topic modeling

**DOI:** 10.1016/j.mex.2022.101650

**Published:** 2022-02-27

**Authors:** Arash Hajikhani, Lukas Pukelis, Arho Suominen, Sajad Ashouri, Torben Schubert, Ad Notten, Scott W. Cunningham

**Affiliations:** a VTT Technical Research Center of Finland, Finland; b Public Policy and Management Institute, Finland; c Fraunhofer Institute for Systems and Innovation Research ISI, Germany; d Maastricht University School of Business and Economics, the Netherland; e Department of Government, University of Strathclyde, United Kingdom; f CIRCLE - Centre for Innovation Research, Lund University, Sweden; g Department of Industrial Engineering, Tampere University, Finland

**Keywords:** Natural language processing, Economic classification scheme, Knowledge transformation, Web scraping

## Abstract

This paper demonstrates a method to transform and link textual information scraped from companies' websites to the scientific body of knowledge. The method illustrates the benefit of Natural Language Processing (NLP) in creating links between established economic classification systems with novel and agile constructs that new data sources enable. Therefore, we experimented on the European classification of economic activities (known as NACE) on sectoral and company levels. We established a connection with Microsoft Academic Graph hierarchical topic modeling based on companies' website content. Central to the operationalization of our method are a web scraping process, NLP and a data transformation/linkage procedure. The method contains three main steps: data source identification, raw data retrieval, and data preparation and transformation. These steps are applied to two distinct data sources.

## Graphical abstract

**Figure:**
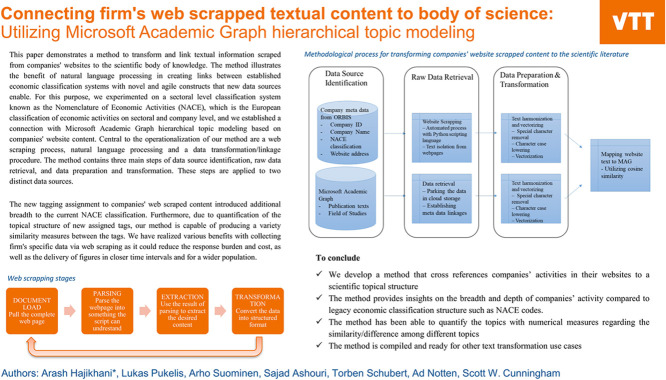

Specifications table

**Table:** 

Subject Area;	Economics and Finance
More specific subject area;	*Informetrics, Scientometrics, Science and Technology Evaluation*
Method name;	*A method for creating a linkage between web scraped company's website content to scientific literature topical structure*
Name and reference of original method;	*Not applicable*
Resource availability;	*Refs.*[1], [2], [3]

The new tagging assignment to companies' web scraped content introduced additional breadth to the current NACE classification. Furthermore, due to quantification of the topical structure of new assigned tags, our method can produce similarity measures between the tags. We have realized various benefits with collecting firm's specific data via web scraping as it could reduce the response burden and cost, as well as the delivery in closer time intervals and for a wider population.

• We develop a method that cross references companies’ activities in their websites to a scientific topical structure.
• The method provides insights on the breadth and depth of companies’ activity compared to legacy economic classification structure such as NACE codes.
• The method has been able to quantify the topics, which demonstrates the similarity/difference among different topics.

## Rationale

Companies typically use their websites to report on their products and services, to present their activities and reference customers, but also to inform their customers and partners about current events related to their business activities [4,5]. Moreover, companies utilize webpages to find new alliances in the value chain, for presenting their profitability and organizational structures to the new potential investors, and for signaling applicable social corporate responsibilities, ethics, and compliance to social audiences or regulators.

Using website data comes with a number of requirements and challenges in terms of data acquisition, data analysis and data validation given the wide range of communication purposes. While extracting relevant information from unstructured or semi-structured textual data from corporate websites can be challenging, it promises a number of benefits, particularly in terms of the granularity, timeliness, scope and cost of collection [6]. Achieving these benefits will also be central to the pursuit of our method.

In addition to simple keyword-based approaches (e.g. measuring the diffusion of standards [7]), approaches with more sophisticated NLP methods have been successfully used to generate web-based firm-level innovation indicators [8]. Our research attempts to add value to the ongoing efforts to utilize data on business and economic oriented activities on a company level and its classifications reallocation. For this purpose, we rely on new data sources and novel indicators. We develop a method to utilize companies' web pages as a data source to retrieve the textual content and then transform it to a data reference point. Rigorous text analytics and natural language processing methods have been performed to map companies' activities directly harvested from their websites. Achieving these outcomes depends upon the use of a hierarchical topic modeling classification cross-compiled with scientific publications.

Use of this topic model enables companies' activities to be compared to a common reference point. Company activities may thereby be embodied within the scientific literature and compared across companies within the same sector. The advantage of mapping companies' website scraped content to a glossary of scientific literature is that it may potentially relate and expand the relevance of companies' claimed activities to science. This effort enhances the classification of companies' activities based on scientific disciplines and enables the discovery of similarities and differences with scientific oriented activities on a topical structured level.

One reason for making this match is that it allows us to disentangle firm activities within NACE categories in more detail. It is well known that even narrow NACE sectors cover very heterogeneous firms. Yet, this heterogeneity is typically hidden within the sector and therefore tends to be ignored in high-level statistical and economic analyses and surveys. Particularly, when it comes to the assessment of firms' capabilities through their products (such as the deployment of digital features in these products), the current use of NACE codes fails in distinguishing of inter-industry activities. For instance, under the NACE code 21 – Manufacturing of pharmaceuticals – we find a diverse pool of companies which among other things included manufacturers of cosmetics, companies that organize clinical trials for various drug candidates, and even sewage treatment companies. This can in many circumstances lead to heavily biased results when used in economic survey. Our results document that although MAG is initially designed for and based on academic texts, it also provides a useful vocabulary for the analyses of economic activities.

## Analytical framework

In order to contribute to this challenge, we both acquire new data sources and methods to offer a systematic path for a fast, reliable and accurate representation of companies' activities while maintaining a structure and harmony. In this method paper we developed a process that incorporates Microsoft Academic Graph (MAG) structured data for Fields of Study (FOS) tagging any textual content. By assigning these categories to company website data and the descriptions of company products, we can classify what a company is doing according to their website content in a granular yet standardized way (as there are over 700 K unique FOS categories). In appendix 1 an example of this transformation is shown.

Based on a methodological pipeline described below the companies' web scraped content is assigned to an equivalent FOS. The scraped texts are pulled into a single corpus for each company, and the TF-IDF scoring is performed on the terms therein. We then construct a vector from the terms and their TF-IDF scores, and next perform a cosine similarity analysis to determine which FOS codes have the highest similarity scores. Due to the weight distribution's long tail, the process considers the 100 most similar FOS codes and associates these with a company. The compiled model and a workable code in Jupyter notebook format with descriptions of the steps are shared.^1^

Because these FOS categories are hierarchically linked, it is possible to easily assess how similar or different FOS categories are to each other. This can be used to go from low-level specific descriptors to high-level categories. Using this hierarchy, opportunities are created to investigate categorizing companies into clusters, isolate and target specific companies, or simply determine how similar different activities/products of the same company are to each other. Moreover, it offers new avenues for identifying firms’ economic activities, innovation activities and firms’ unique set of capabilities. The code for assessing the FOS codes similarity is compiled and shared as part of this work.^2^ The linking and classifying companies' website content to FOS codes can transform high dimensional textual information into technology and science-related activities. Once a companies' activities are offered in higher granularity and harmonized, this offers the possibility to create additional indicators with more thematic orientation (i.e., AI-related activities).

## Data description

Our findings result from web scraping 96,921 companies. These companies are drawn from all European companies within the high technology, and medium-high technology categories of NACE codes, as assigned by the European Union. Each of the constituent companies which are scraped can be categorized by means of 4-digit NACE codes. There are 90 distinct NACE codes in this sample. The method detailed below demonstrates the categorization of this data using MAG field of study codes. A single case for analyzing industrial structure is the 4-digit NACE code, or higher-level aggregations of NACE such as the 2-digit categorization of firms.

### Method details: the four steps

**Fig. 1:**
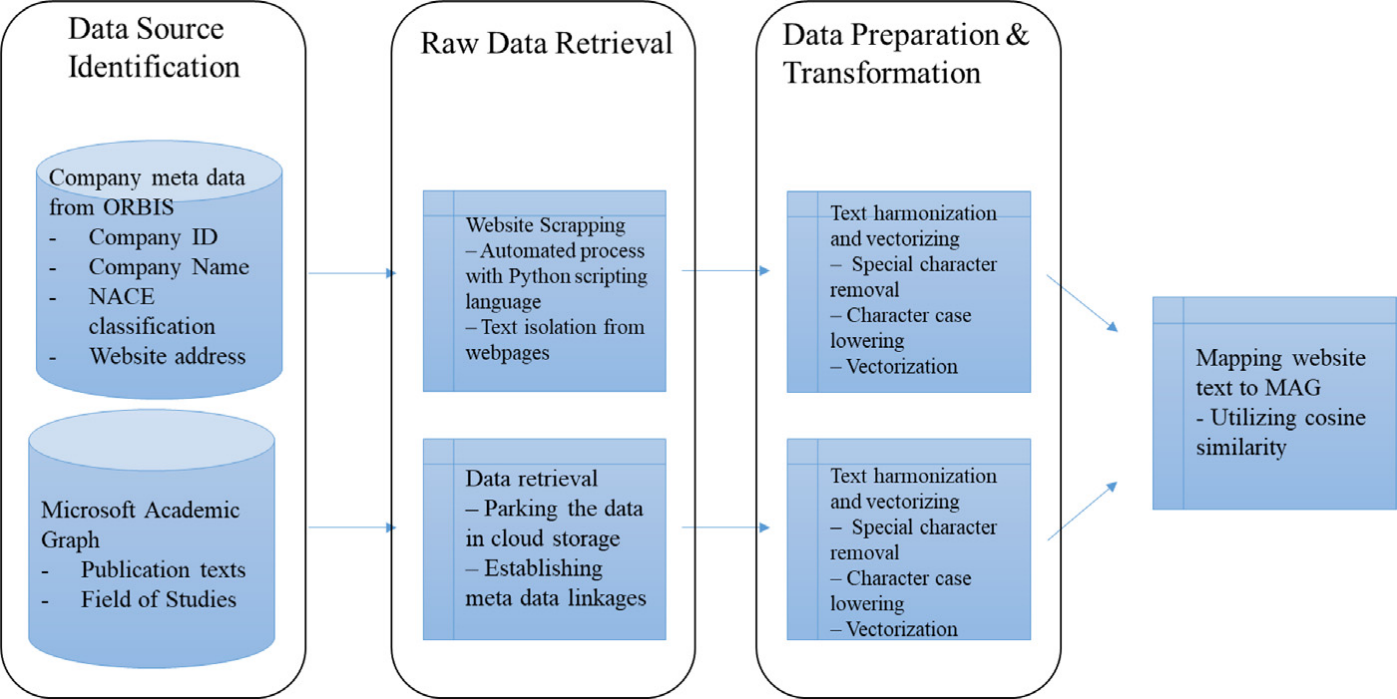
Methodological process for transforming companies' website scrapped content to the scientific literature.

Fig. 1 illustrates the methodological process for transforming the textual content scraped from companies' websites to their equivalent in scientific literature.

We present our initial finding with a case study of 96,921 European Union registered companies from whose websites we scraped data [9,10]. The data was retrieved, from the company websites, from December of 2020 to August of 2021. We discuss the four-step process used to create the results in the following sections. Finally, we present the network structure created using NACE and MAG based codes and discuss the implications of their relationship.

### Data source identification

Company-related data is fetched from a proprietary database on firm data from Bureau van Dijk (BvD). Their database “Orbis” contains business records for 415,094,925 firms (as of November 2021), of which 302,254,350 were marked as active (https://orbis.bvdinfo.com). The company-level variables are available from the Orbis database. In contrast to other company databases, such as Datastream, which offers information on stock listed companies only, Orbis covers small and medium-sized companies as well. The Orbis company data contain website addresses of companies that are necessary data points for our experiment, serving as the seed value for the web scraping. For this experiment, a sample of 183,161 companies was identified as high tech and medium-high tech, according to the Eurostat aggregation of manufacturing industries based on its technological intensity and using the NACE revision 2 coding for high and medium-high tech industries.

For creating an alternative method to capture companies' activities in more breadth and depth, we utilized topics or Fields of Study (FOS) from Microsoft Academic Graph (https://academic.microsoft.com/topics). Microsoft Academic Graph (MAG) is a large and heterogeneous graph comprising more than 120 million publications and related bibliometric metadata. As of today, MAG is the largest publicly available dataset of scholarly publications and the largest dataset of open citation data. MAG data models scholarly communication activities which consist of six types of entities — publications, authors, institutions (affiliations), venues (journals and conferences), fields of study and events (specific conference instances); and the relations between these entities such as cross-citations and authorship. The coverage of MAG data is illustrated in Table 1 (as of November 2021). The relations between the entities are described in more detail in [3,11], and the MAG database scheme can be accessed from [2].

**Table 1:** MAG data description.

MAG Entity Descriptive	
Publications	268,618,709
Authors	279,177,391
Field of Studies (FOS)	714,595
Conferences	4550
Journals	49,062
Institutions	27,057

MAG uses a curated topic model. These are grouped into fields of study. These fields of study are hierarchical in nature, grouping specific fields of study under larger, more generic fields of study. A description of these topics is available on the Microsoft Academic Graph website, and the underlying data can be downloaded into local cloud accounts using the Azure platform.

## Raw data retrieval

The data platform utilizes a “hybrid” design with part of the infrastructure being located on-site, and the other part in the “cloud” (MS Azure Cloud Platform). The “cloud” part of the data platform was created by allocating a virtual machine and setting up the required resources: Jupyter Hub and PostgreSQL database there.

**Fig. 2:**
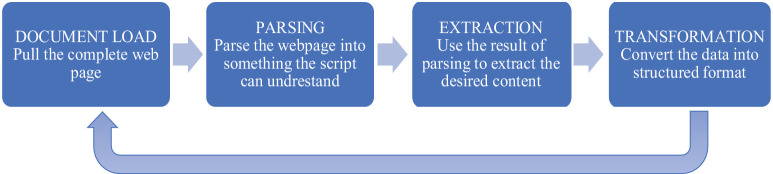
Web scrapping stages.

Companies' website addresses were obtained from Bureau van Dijk “Orbis” in batches (xlsx format). The URL for each company is then used as a seed value in textual format in the content retrieval pipeline designed in Python. Scraping is performed by using Python libraries provided by HTMLParser and Beautiful Soup. The website links crawled using the Beautiful Soup package are then categorized and stored in database according to the category assigned. The process step performed by the web crawler are: (1) Enter a URL and use a HTTP request to access the URL (2) Fetch all the contents in the URL and parse the textual data (3) Store the data in any desired database. (4) Enqueue all the URLs in a page. (5) Use the URLs in the queue and repeat from process 1 Fig. 2. illustrates the process.

MAG and its associated data is installed on the Microsoft Azure storage infrastructure. The Microsoft website explains detailed step-by-step instructions for setting up one-time or automatic provisioning of Microsoft Academic Data (MAKES/MAG) to an Azure blob storage account.^3^ For the assignments of FOS fields to publication's text, we followed the procedure explained in Wang et al. [2].

## Data preparation and transformation

The data preparation and transformation for the process includes using the two data sources as described above. First, the analysis uses the web scraped textual data from the company websites. Second, the process uses the publication text from Microsoft Academic associated with different MAG FOS codes. In practice, the objective is to create a quantitative representation of documents in the web scraped data and publication data per FOS code. Then, using the information of publications association with FOSs, the web scraped data infers an association with the FOS codes.

The analysis uses all of the publications in MAG and the FOS IDs associated with the publications. By merging the publications by individual FOS codes, a single corpus was created for each unique FOS code. This means that each of the 700 thousand FOS codes are now associated with a separate representative corpus. Each of the corpora are pre-processed, and a vector representation is created using Term Frequency - Inverse Document Frequency (TF-IDF) scores. From the vector representations, 1000 highest weighted terms across the corpora are selected for representing a specific topic. This resuls in a matrix representation. In the matrix the rows represent the individual FOSes while the columns represent the terms. The cell values are the TF-IDF scores for each term in that specific FOS.

For the web scraped text, the objective is similarly to create a vector representation. Data retrieved from each website was independently pre-processed and vectorized using the TF-IDF approach. Terms not included in the matrix (built from FOS codes), were excluded from the company website vector representation to allow for similarity measures to be built. Having two matrix representations of content allows for the comparison and assignment of relevant topics to the websites.

The pre-processing for all data involves cleaning procedures (e.g. stop words removal, non-alphanumeric characters removal, stemming and lowercase transformation) applied to harmonize and increase the consistency of the text.^4^ Our web scraping pipeline was in compliance with GDPR as it excluded any text collection from the “contact us” section of the websites and avoided any name and personal detail collection. For natural language processing (NLP) to work it requires transforming natural language (text) into a vector representation. Text vectorization techniques, namely TF-IDF, bag of words and vectorization, are very popular choices for classification algorithms, and can help convert textual information to numeric feature vectors. Therefore, to quantify and convert text into a numerical representation in documents, we compute a weight for each phrase that signifies the importance of the phrase in the document and corpus. The TF-IDF method is a widely used technique in Information Retrieval and Text Mining [1]. TF-IDF is a weighting procedure that tries to evaluate the relevance of terms in the document corpus. As the term implies, TF-IDF calculates values for each phrase in a document through an inverse proportion of the frequency of the phrase in a particular document to the percentage of the documents that the phrase appears in. The below is the formula for how to compute the term weighting by TF-IDF:

(1) $$w_{jk}=tf_{jk}\times idf_{j}$$

$w_{jk}=$ phrase weight of phrase j in document k

$tf_{jk}=$ the number of phrases j that occur in document k

here variable $idf_{j}$ is the inverse document frequency of phrase j as derived in the following equation

(2) $$idf_{j}=\text{lo}g_{2}\left(\frac{n}{df_{j}}\right)$$

n= the total number of documents in the document set

$df_{j}=$ the number of documents containing the phrase j in the document set

Using the transformation it's possible to obtain a similarity measure of any pair of vectors which yields a measurement that quantifies the similarity between two or more vectors. In practice, the process uses cosine similarity,^5^ which is a measure of similarity which can be calculated using any non-zero vectors using a dot product. The cosine similarity of TF-IDF is a credible means of assigning website content to the corresponding MAG FOS IDs. The proposed approach enables us to obtain structured fields of study data (FOS IDs) for each of the high dimensional text vectors that are scraped from company websites.

## Conclusion

The process described here resulted in the identification and mapping of over 10,000 unique FOS codes to this sample of scraped high-technology web pages. There may well be more FOS codes which could be identified, as these results used only the top 100 codes by similarity as identified on each web pages. This demonstrates the diversity of knowledge in this sample of high-technology European firms.

The need to obtain more granular data on the activities of companies is evident. The shortcomings of the existing industry classifications (NACE codes) in this respect are clear, and new classification methods should be considered to capture the breadth and depth of companies' activities, as well as the dynamic changes to their industrial and economic focus. The process described in this paper enables more systematic and more detailed investigation into actual corporate activities. In turn this might create a more accurate and more dynamic industrial classification system. Companies as well as the public sector will benefit from the resultant outputs.

## Direct submission or co-submission

Direct submission.

## Funding source

This project has received funding from the European Union's Horizon 2020 research and innovation program under grant agreement No 870822.

## Declaration of Competing Interest

None.

## Figures and Tables

**Table 2:** Examples of FOS tagging.

Text (from a publication abstract)		FOS tags with normalized percentage scale ratio of dominance (0:not similar - 100:similar)	
Retinitis pigmentosa (RP) is an inherited retinal dystrophy caused by the loss of photoreceptors and characterized by retinal pigment deposits visible on fundus examination. Prevalence of non syndromic RP is approximately 1/4000. The most common form of RP is a rod-cone dystrophy, in which the first symptom is night blindness, followed by the progressive loss in the peripheral visual field in daylight, and eventually leading to blindness after several decades. Some extreme cases may have a rapid evolution over two decades or a slow progression that never leads to blindness. In some cases, the clinical presentation is a cone-rod dystrophy, in which the decrease in visual acuity predominates over the visual field loss. RP is usually non syndromic but there are also many syndromic forms, the most frequent being Usher syndrome. To date, 45 causative genes/loci have been identified in non syndromic RP (for the autosomal dominant, autosomal recessive, X-linked, and digenic forms). Clinical diagnosis is based on the presence of night blindness and peripheral visual field defects, lesions in the fundus, hypovolted electroretinogram traces, and progressive worsening of these signs.		{“Progressive visual loss”: 32, “Visual field loss”: 28, “Cone-Rod Dystrophy”: 28, “Complete Blindness”: 28, “Dystrophy”: 27, “Visual field”: 27, “Meridian (perimetry, visual field)”: 26, “Functional visual loss”: 26, “Visual field test”: 26, “Total blindness”: 26, “Visual field testing”: 25, “Blindness”: 25, “Quadrantanopia”: 24, “Visual Disturbance”: 24, “Visual defects”: 24, “Sudden visual loss”: 24, “Hemianopsia”: 24, “Anterior Visual Pathway”: 23, “Visual Physiology”: 23, “Visual deficit”: 23, “Visual changes”: 23, “Retinal Dystrophies”: 22, “Goldmann perimetry”: 22, “Visual Disorders”: 22, “Visual prosthesis”: 22, “Transient blindness”: 22, “Cone dystrophy”: 21, “Retinitis pigmentosa”: 21, “Visual structure”: 21, “Visual research”: 21, “Visual symptoms”: 21, “Gene therapy of the human retina”: 21, “Visual system”: 21, “Automated static perimetry”: 21, “Visual abnormalities”: 21, “Visual approach”: 20, “Visual phenomena”: 20, “Visual rhetoric”: 20, “Visual Manifestations”: 20, “Gaze-contingency paradigm”: 20, “Peripheral vision”: 20, “Optic disk pallor”: 20, “Visual space”: 20, “Visual processing”: 20, “Central vision”: 20, “Vision for perception and vision for action”: 20, “Visual rehabilitation”: 20, “Macular dystrophy”: 19, “Cortical blindness”: 19, “Visual capture”: 19,	
Molecular diagnosis can be made for some genes, but is not usually performed due to the tremendous genetic heterogeneity of the disease. Genetic counseling is always advised. Currently, there is no therapy that stops the evolution of the disease or restores the vision, so the visual prognosis is poor. The therapeutic approach is restricted to slowing down the degenerative process by sunlight protection and vitaminotherapy, treating the complications (cataract and macular edema), and helping patients to cope with the social and psychological impact of blindness. However, new therapeutic strategies are emerging from intensive research (gene therapy, neuroprotection, retinal prosthesis).		“Biased Competition Theory”: 19, “Visual behavior”: 19, “Visual control”: 19, “Visual sensory”: 19, “Visual threshold”: 19, “Visual language”: 19, “VISUAL TRAINING”: 19, “Nyctalopia”: 19, “Visual technology”: 19, “Central scotoma”: 19, “Slow progression”: 19, “Visual communication”: 19, “Visual snow”: 19, “Visual sociology”: 18, “Leber's congenital amaurosis”: 18, “N2pc”: 18, “Change blindness”: 18, “Tunnel vision”: 18, “Visual hierarchy”: 18, “Neuro-ophthalmology”: 18, “Blindsight”: 18, “Humphrey visual field”: 18, “Leber congenital amaurosis”: 18, “Optic neuropathy”: 18, “Goldmann perimeter”: 18, “Visual testing”: 18, “Posterior ischemic optic neuropathy”: 18, “Visual N1”: 17, “Ischemic optic neuropathy”: 17, “Visual thinking”: 17, “Visual culture”: 17, “Tangent screen”: 17, “Visual Suppression”: 17, “Bilateral blindness”: 17, “Retinal degeneration”: 17, “Visual Objects”: 17, “Legal blindness”: 17, “Visual reasoning”: 17, “Vision therapy”: 17, “Visual estimation”: 17, “Visual Ergonomics”: 17, “Visual angle”: 17, “Visual impairment”: 16, “Homonymous hemianopsia”: 16, “PDE6B”: 16, “RPE65”: 16, “Visual phototransduction”: 16, “Molecular therapy”: 16, “Visual appearance”: 16, “Visual contrast”: 16}	


Company name and website	NACE codes	Company's website keywords	FOS tags with normalized percentage scale ratio of dominance (0:not similar - 100:similar)


“WOLFVISION GMBH”, “www.wolfvision.at”	NACE Rev. 2 main section: C-Manufacturing Core code: 2751, Manufacture of electric domestic appliances	{“hybrid learning”: 125, “screen sharing”: 108, “collaborative learning”: 85, “learning collaborative”: 68, “on-screen display”: 68, “remote management”: 64, “educational institution”: 50, “student education”: 50, “county court”: 38, “imaging quality”: 37, “web conferencing”: 36, “co working”: 33, “public research”: 31, “class collaboration”: 28, “firmware version”: 28, “image storage”: 28, “single center”: 27, “core product”: 26, “additional feature”: 24, “lecture capture”: 22, “online teaching”: 21, “matrix solution”: 21, “light system”: 21, “teaching staff”: 20, “health science”: 20, “classroom teaching”: 19, “core system”: 18, “provide access”: 16, “administration of justice”: 16, “bring your own device”: 15}	{“Collaboration tool”: 22, “Presentation logic”: 19, “Mass collaboration”: 18, “Virtual collaboration”: 18, “Social collaboration”: 17, “Distributed collaboration”: 17, “Meeting Request”: 17, “Variable presentation”: 16, “Electronic meeting system”: 15, “Meeting Reports”: 15, “Collaborative software”: 14, “Hybrid system”: 14, “Online document”: 14, “Presentation Manager”: 14, “Mobile collaboration”: 13, “Whiteboard”: 13, “Fixed wireless”: 13, “Face Presentation”: 13, “Presentation layer”: 13, “Technical Presentation”: 13, “Wireless site survey”: 13, “Sales presentation”: 13, “Scientific collaboration network”: 13, “Collaboration”: 12, “Meeting Abstracts”: 12, “Wi-Fi array”: 12, “Wireless Internet Protocol”: 12, “Disease Presentation”: 12, “Wireless network interface controller”: 12, “Hybrid material”: 12, “Bring your own device”: 11, “Wireless LAN controller”: 11, “Wireless intrusion prevention system”: 11, “Wireless grid”: 11, “Multimedia”: 11, “Hybrid”: 11, “Online presence management”: 11, “Transverse presentation”: 11, “Motorola Canopy”: 11, “Authoring system”: 11, “Online participation”: 11, “Online learning community”: 11, “Content creation”: 11, “CAPWAP”: 11, “Content management”: 11, “Web annotation”: 11, “Hybrid intelligent system”: 11, “Meeting Material”: 11, “Online research methods”: 11,
			“Municipal wireless network”: 11, “Computer-supported collaborative learning”: 11, “Computer-supported cooperative work”: 11, “Asynchronous learning”: 10, “Team meeting”: 10, “Online discussion”: 10, “Wireless security”: 10, “Document engineering”: 10, “Document management system”: 10, “Wireless Application Protocol”: 10, “Web content management system”: 10, “Vision document”: 10, “Web document”: 10, “Learning Management”: 10, “Application sharing”: 10, “Computer-mediated communication”: 10, “Quality documents”: 10, “Online computer”: 10, “Web content”: 10, “Web 2.0″: 10, “Cross-presentation”: 10, “Online community”: 10, “Collaborative learning”: 10, “Online help”: 10, “Educational technology”: 10, “Media space”: 10, “Certified Wireless Network Administrator”: 10, “Wireless”: 10, “Summary (document)”: 10, “Vertex Presentation”: 10, “Synchronous learning”: 10, “Blended learning”: 10, “Wireless USB”: 10, “Hybrid power”: 10, “Supply chain collaboration”: 10, “Virtual learning environment”: 10, “Wireless WAN”: 10, “Collaborative editing”: 10, “Teleconference”: 10, “Computer-assisted web interviewing”: 10, “Classroom management”: 10, “Collaborative engineering”: 10, “Online identity”: 10, “Hybrid Bond”: 9, “Source document”: 9, “Hybrid zone”: 9, “Living document”: 9, “Fetal Presentation”: 9, “Online degree”: 9, “Open classroom”: 9, “Wi-Fi”: 9}

## Footnotes

1 The code can be accessed from Github at https://github.com/arash-hajikhani/Bigprod_FOS/blob/main/Text-to-FOS-Similarity.ipynb.

2 The code for FOS similarity assessment can be accessed from Github at https://github.com/arash-hajikhani/Bigprod_FOS/blob/main/FOS_Similarity.ipynb.

3 Please refer to https://docs.microsoft.com/en-us/academic-services/graph/get-started-setup-provisioning.

4 For text cleaning, harmonizing and structuring the following python packages was utilized: Scrapy, NLTK 3.5, Pandas 1.1.3, Numpy 1.19.2 and re 2.2.1.

5 To read more on calculation of cosine similarity, refer to Cosine Similarity chapter in Han et al. [12].
